# First delirium episode in Parkinson’s disease and parkinsonism: incidence, predictors, and outcomes

**DOI:** 10.1038/s41531-021-00234-2

**Published:** 2021-10-11

**Authors:** Samantha Green, Sarah L. Perrott, Andrew McCleary, Isobel Sleeman, Jodi Maple-Grødem, Carl E. Counsell, Angus D. Macleod

**Affiliations:** 1 Division of Applied Health Sciences, University of Aberdeen, Aberdeen, UK; 2 Ninewells Hospital, Dundee, UK; 3 The Norwegian Centre for Movement Disorders, Stavanger University Hospital, Stavanger, Norway; 4 Department of Chemistry, Bioscience and Environmental Engineering, University of Stavanger, Stavanger, Norway

**Keywords:** Epidemiology, Outcomes research, Parkinson's disease

## Abstract

To define the incidence, predictors and prognosis of the first hospital delirium episode in Parkinson’s disease (PD) and atypical parkinsonism (AP), we identified the first hospital episode of delirium after diagnosis in the Parkinsonism Incidence in North-East Scotland (PINE) study, a prospective community-based incidence cohort of parkinsonism, using chart-based criteria to define delirium. Of 296 patients (189=PD, 107=AP [dementia with Lewy bodies, progressive supranuclear palsy, multiple system atrophy, vascular parkinsonism]), 152 developed delirium (PD = 98, AP = 54). Incidence of first hospital delirium episode per 100 person years was 8.1 (95% confidence interval [CI] 6.6–9.9) in PD and 18.5 (95% CI 13.9–24.7) in AP. Independent predictors of delirium were atypical parkinsonism (Hazard ratio [HR] vs PD = 2.83 [95% CI 1.60–5.03], age in PD but not in AP (HR for 10-year increase 2.29 [95% CI 1.74–3.02]), baseline MMSE (HR = 0.94 [95% CI 0.89–0.99]), *APOE* ε4 in PD (HR 2.16 [95% CI 1.15–4.08]), and *MAPT* H1/H1 in PD (HR 2.08 [95% CI 1.08–4.00]). Hazards of dementia and death after delirium vs before delirium were increased (dementia: HR = 6.93 [95% CI 4.18–11.48] in parkinsonism; death: HR = 3.76 [95% CI 2.65–5.35] in PD, 1.59 [95% CI 1.04–2.42] in AP). Delirium is a common non-motor feature of PD and AP and is associated with increased hazards of dementia and mortality. Whether interventions for early identification and treatment improve outcomes requires investigation.

## Introduction

Delirium is a common acute neuropsychiatric syndrome involving changes in consciousness, cognition or perception. Delirium is under-recognised despite being associated with increased risk of falls, cognitive decline, morbidity, and mortality^1^. It is an important non-motor feature that has hitherto been neglected in Parkinson’s disease and other forms of parkinsonism.

While it is widely believed that delirium is more common in parkinsonism than in the general ageing population, there is currently limited evidence for its risk factors, incidence, prevalence and prognosis^2^. Previous research has explored the development of delirium in PD within restricted environments such as neurology wards^3^ and emergency admissions^4^, but only one small study has investigated delirium in general hospital admissions with a robust definition of delirium^5^. No study has examined the development of delirium over the life course of PD or parkinsonism so there are no previous estimates of its incidence. The few studies that have investigated the predictors of delirium in PD have been small^5,6^. There are few data on outcomes of delirium in PD, with only one very small study of delirium in PD reporting an increased risk of dementia, motor impairment and mortality^7^. We are unaware of data on delirium in atypical parkinsonism other than its prevalence in dementia with Lewy bodies (DLB)^8^.

We aimed to identify (i) the incidence of, (ii) the risk factors for, and (iii) the outcomes after, the first hospital episode of delirium in both PD and atypical parkinsonism.

## Results

### Description of participants

**Fig. 1:**
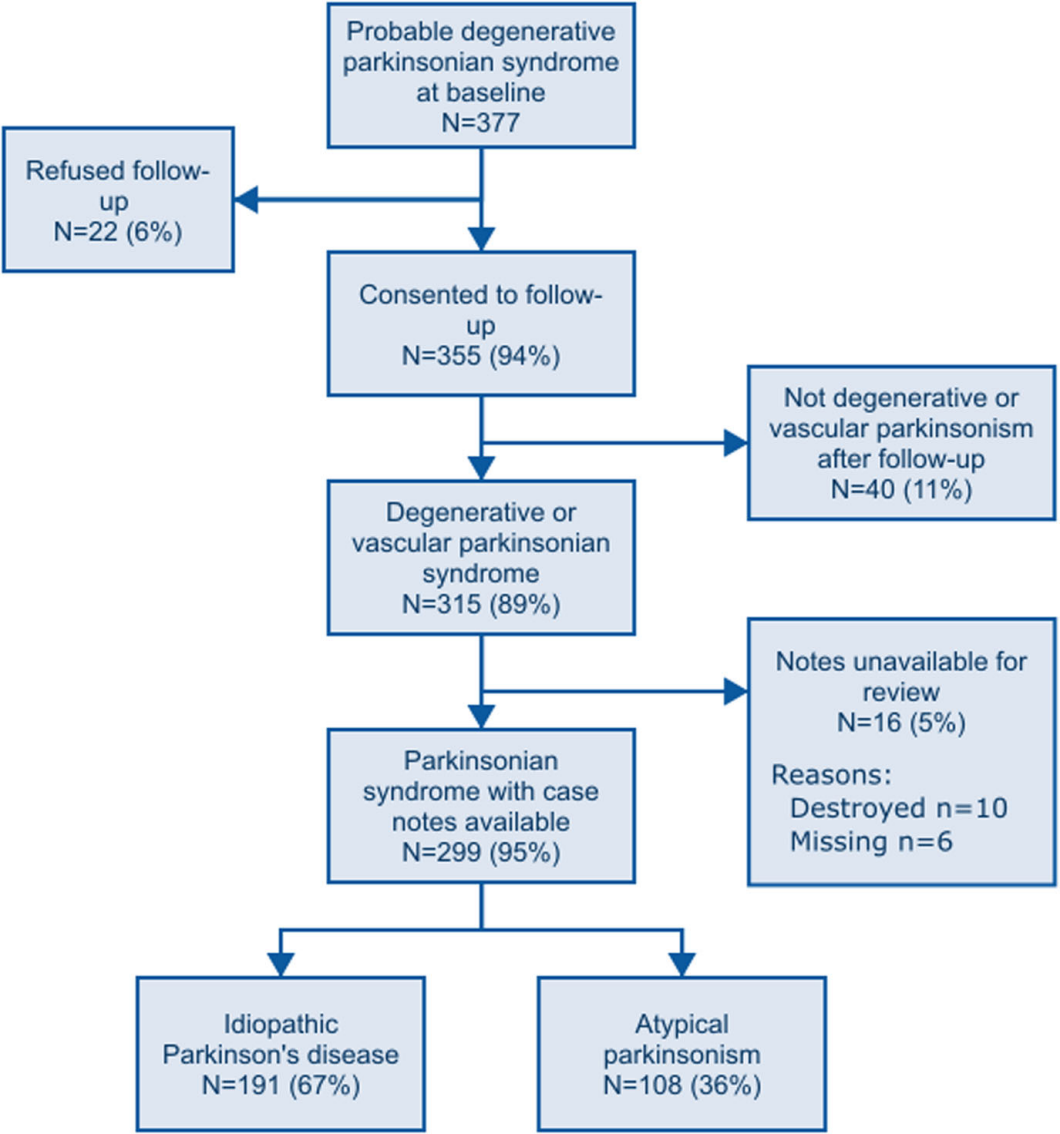
Flowchart. Flowchart of study participation with reasons for non-participation and exclusion from analysis.

Of 377 with suspected incident parkinsonism, 355 (94%) consented to follow-up and were recruited to the study (Fig. 1). Of these 315 (89%) were confirmed to have a degenerative or parkinsonian syndrome after follow-up. Of these, 299 (95%) had case notes available for review. 191 had idiopathic PD (mean age at diagnosis 72.6 years, 40% female) and 108 had atypical parkinsonism (mean age at diagnosis 78.8 years, 36% female). Baseline characteristics are shown in Table 1. There were no important differences in baseline characteristics between those with and without case notes available. Three patients moved away from the area before the development of delirium and were lost to follow-up for this outcome. Those with case notes available but who were not admitted to the hospital were included in the analysis (*N* = 35 [12%]; mean age at diagnosis = 73.0 [SD 11.7], 37% female).

**Table 1:** Participants’ baseline characteristics.

	All PINE patients with parkinsonism (*N* = 315)	Patients with case notes available for review (*N* = 299)	Idiopathic PD (with case notes available) (*N* = 191)	Atypical parkinsonism (with case notes available) (*N* = 108)
Mean age at diagnosis (SD)	74.8 (9.8)	75.0 (9.6)	72.9 (10.1)	78.7 (7.3)
Female, *N* (%)	120 (38.1)	111 (37.5)	74 (38.7)	39 (36.1)
DepCat score^a^	2.8 (1.6)	2.8 (1.6)	2.8 (1.6)	2.8 (1.6)
Diagnosis, *N* (%)				
Idiopathic PD	200 (63.5)	191 (63.9)		
PSP/CBD	30 (9.5)	29 (9.7)		
MSA	11 (3.5)	11 (3.7)		
DLB	37 (11.7)	36 (12.0)		
Vascular parkinsonism	33 (10.5)	28 (9.4)		
Dementia with associated parkinsonism	4 (1.3)	4 (1.3)		
Median baseline MMSE^b^ (IQR)	28 (24-29)	28 (24-29)	29 (28-30)	23 (17-26)
Mean baseline UPDRS (SD)	28.2 (13.2)	28.0 (12.9)	25.1 (11.7)	33.1 (13.5)
Median baseline Charlson co-morbidity index (IQR)	1 (0-2)	1 (0-2)	1 (0-2)	1 (0-2)
Median baseline Schwab & England score (IQR)	80 (60-90)	80 (60-90)	90 (80-95)	60 (40-80)
Mean baseline Hoehn & Yahr stage (SD)	2.6 (1.0)	2.6 (1.0)	2.3 (0.8)	3.2 (1.1)
APOE ε4 carrier, n/N (%)	35/122 (29)	35/120 (29)	35/120 (29)	N/A
GBA variant carrier, n/N (%)	11/118 (9)	11/116 (9)	11/116 (9)	N/A
MAPT H1/H1, n/N (%)	88/122 (72)	86/120 (72)	86/120 (72)	N/A

*PD* Parkinson’s disease, *PSP* progressive supranuclear palsy, *MSA* multiple system atrophy, *DLB* dementia with Lewy bodies, *MMSE* mini-mental state examination, *UPDRS* unified Parkinson’s disease rating scale, *APOE* Apolipoprotein E, *GBA* glucocerebrosidase, *MAPT* microtubule-associated protein tau, *SD* standard deviation, *IQR* inter-quartile range.

^a^DepCat score is a postcode-derived socioeconomic status indicator. 1 = least deprived, 6 = most deprived.

^b^26 cases with notes available had missing MMSE at baseline.

### Incidence of first delirium episode

**Fig. 2:**
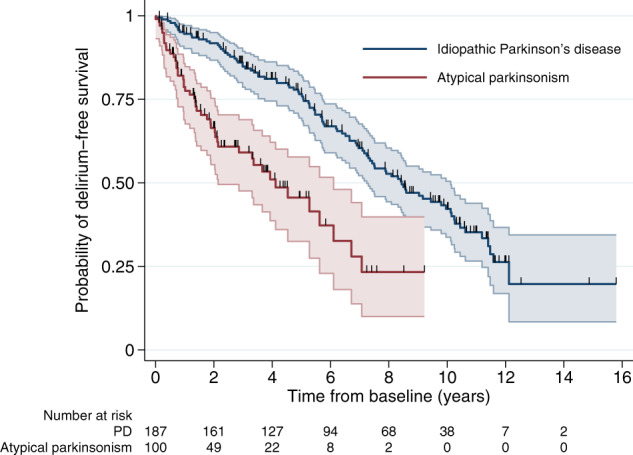
Development of delirium. Kaplan–Meier probabilities of survival free of delirium (first hospital episode) in PD and atypical parkinsonism.

Over 1896 person years of follow-up, 152 patients developed at least one hospital delirium episode (PD = 98, atypical parkinsonism = 54). Ten patients (8 with atypical parkinsonism, 2 with PD) had delirium during an admission in which the parkinsonian syndrome was diagnosed and were excluded from further analyses. Details of the hospital admissions and the features of delirium extracted from case records are in Supplementary Data 1. Median time from diagnosis to first delirium episode was 8.4 years (95% confidence interval [CI] 7.2–10.1) in PD and 4.1 years (95% CI 2.1–6.1) in atypical parkinsonism (Fig. 2). The incidence rate of first hospital delirium episode per 100 person years was 8.2 (95% CI 6.7–10.0) in PD and 18.4 (95% CI 13.8–24.5) in atypical parkinsonism. The incidence rates per 100 person years in patients without dementia were 6.5 (95% CI 5.2–8.3) in PD and 12.6 (7.7–20.5) in atypical parkinsonism. Data on incidence of first hospital delirium episode in individual syndromes are given in Table 2. All those with a documented diagnosis of delirium made by an experienced clinician (*N* = 45; 41 a geriatrician, 3 a psychiatrist, 1 a neurologist) met the three study criteria for diagnosis of delirium also. Infection was the commonest cause of delirium (51% of cases). Other causes are listed in Table 3. In patients with delirium the mean number of hospital admissions from diagnosis of parkinsonism until the development of delirium was 2.5.

**Table 2:** Incidence of first hospital episode of delirium in individual parkinsonian syndromes.

	PD (*N* = 191)	Dementia with Lewy bodies (*N* = 36)	Progressive supranuclear palsy/corticobasal degeneration (*N* = 29)	Multiple system atrophy (*N* = 11)	Vascular parkinsonism (*N* = 28)
Cases of delirium, N (%)	98 (51)	21 (58)	12 (41)	5 (45)	14 (50)
Person years of follow-up	1198.1	74.5	82.2	35.3	53.3
Median time in years from diagnosis to first delirium episode (95% CI)	8.4 (7.2–10.1)	2.1 (1.0–6.7)	NR	5.3 (0.8–ND)	3.7 (1.9–ND)
Incidence rate of first hospital delirium episode per 100 person years (95% CI)	8.2 (6.7–10.0)	25.5 (16.3–40.0)	12.2 (6.5–22.6)	14.1 (5.9–34.0)	20.6 (11.4–37.2)

Four patients with dementia-associated parkinsonism excluded from this Table 2 participants with PD, 2 with DLB, 2 with PSP, and 3 with vascular parkinsonism excluded from median time and incidence rate analysis because they had delirium during an hospital admission during which they were diagnosed with a parkinsonian syndrome.

*CI* confidence interval, *ND* not defined, *NR* not reached.

**Table 3:** Causes of hospital delirium episodes in parkinsonism.

Main underlying cause of delirium	Frequency (%) *N* = 152
Infection	80 (53)
Fracture	16 (11)
Gastrointestinal illness	10 (7)
Neurological illness	7 (5)
Drugs	6 (4)
Elective surgery	5 (3)
Cardiac illness	4 (3)
Respiratory illness	4 (3)
Other	6 (4)
Unclear	14 (9)

### Baseline predictors of delirium

Baseline predictors of delirium from the multivariable Cox model are shown in Table 4. Atypical parkinsonism was associated with higher hazards of delirium than PD (HR = 2.83, 95% CI 1.60–5.03). Age was associated with delirium in PD (HR for 10-year increase in age = 2.29, 95% CI 1.74–3.02) but not in atypical parkinsonism. Lower baseline MMSE was also associated with increased hazards of delirium (HR = 0.94, 95% CI 0.89–0.99) and there was borderline evidence for an association between worse socioeconomic status and delirium (HR for DepCat score 1.10 (95% CI 1.00–1.23). Sex, baseline UPDRS, and Charlson score did not predict delirium in the main analysis. In the secondary analysis in patients without dementia, the associations of these prognostic factors were similar except that MMSE was no longer a significant prognostic factor and DepCat score was more-strongly associated with delirium (HR 1.21, 95% CI 1.06–1.38). In the analysis of genetic predictors in PD only, the *MAPT* H1/H1 haplotype was associated with increased risk of delirium (HR 2.08, 95% CI 1.08–4.00) and *APOE* ε4 carrier status was associated with increased risk of delirium (HR 2.16, 95% CI 1.15–4.08). Although UPDRS was significantly associated with delirium in the subset with PD and genetic data (HR 1.34, 95% CI 1.04–1.73) an interaction between UPDRS and diagnosis in the main model was not significant.

**Table 4:** Baseline predictors of delirium in parkinsonism.

Baseline predictor	All patients *N* = 287		PD patients with genetic data *N* = 115	
	Hazard ratio (95% CI)	*P*-value	Hazard ratio (95% CI)	*P*-value
Atypical parkinsonism vs PD^a^	2.83 (1.60–5.03)	<0.001		
Age (10-year increase)				
In PD	2.29 (1.74–3.02)	<0.001	2.35 (1.64–3.36)	<0.001
In atypical parkinsonism	1.27 (0.84–1.91)	0.26		
Sex	1.16 (0.82–1.66)	0.42	1.43 (0.80–2.53)	0.22
DepCat	1.10 (1.00–1.23)	0.06	1.17 (0.98–1.39)	0.08
Charlson score	1.07 (0.94–1.22)	0.30	1.15 (0.92–1.44)	0.23
UPDRS part 3 (10-point increase)	1.04 (0.87–1.24)	0.68	1.34 (1.04–1.72)	0.02
MMSE (1-point increase)	0.94 (0.89–0.99)	0.04	1.00 (0.85–1.18)	0.98
GBA variant carrier			0.82 (0.30–2.20)	0.69
MAPT H1/H1			2.08 (1.08–4.00)	0.03
APOE ε4 carrier			2.16 (1.15–4.08)	0.02

Mulitvariable Cox regression.

*DepCat* deprivation score (higher DepCat score implies worse socioeconomic status), *MMSE* mini-mental state examination, *UPDRS* Unified Parkinson’s Disease Rating Scale, *APOE* Apolipoprotein E, *GBA* glucocerebrosidase, *MAPT* microtubule-associated protein tau.

^a^Age re-parameterised so that this hazard ratio represents the increased hazards in atypical parkinsonism vs PD at age = 70.

139 patients with delirium (91%) had MMSE documented within the year preceding delirium. The median (IQR) MMSE documented in the last year before delirium was 26 (23–28) in PD and 19 (15–26) in atypical parkinsonism. The development of dementia (as a time-varying covariate) was associated with an increased risk of delirium (HR 1.94, 95% CI 1.29–2.92; *p* = 0.002). In this model, with adjustment for dementia, diagnosis (atypical parkinsonism vs PD) was no longer significant (HR 1.30, 95% CI 0.79–2.14).

### Predictors of delirium at time of hospital admission

105 matched delirium/no delirium pairs were identified (72 pairs with PD, 33 with atypical parkinsonism) (Table 5). 26 participants with PD and 21 with atypical parkinsonism did not have a match without delirium. The significant predictors of delirium in the multivariable case-control analyses were hospital admission due to infection (OR versus other medical cause = 7.88, 95% CI 1.61–38.49), admission due to fracture (OR vs other medical cause = 7.36, 95% CI 2.27–23.87), admission due to other surgical causes (OR versus other medical causes = 0.16 (95% CI 0.03–0.93) and presence of dementia before admission (OR 5.99, 95% CI 1.86–19.24). Several admissions due to other surgical causes were elective minor procedures, contributing to the lower risk. The emergency vs elective variable was excluded from the main model because of collinearity with the cause of admission variable. In a model with this variable and not the cause of admission variable, emergency admissions were associated with a higher risk of delirium than elective admissions (OR 7.36, 95% CI 1.98–27.4). No other risk factor at hospital admission was associated with delirium.

**Table 5:** Nested case-control analysis: participant characteristics and associations with delirium.

Variable	Delirium *N* = 105	Not delirium *N* = 105	Association with delirium			
			Univariable analyses		Multivariable analyses	
			OR (95% CI)	*P*-value	OR (95% CI)	*P*-value
Age at baseline in years	77.5 (6.2)^a^	77.4 (6.3)^a^	1.16 (0.84–1.59)	0.36	0.96 (0.61–1.53)	0.87
Male sex	69 (66)^b^	69 (66)^b^	NA		NA	
Diagnosis			NA		NA	
PD	72 (69)^b^	72 (69)^b^				
Atypical parkinsonism	33 (31)	33 (31)				
Disease duration in years	2.6 (1.0–5.8)^c^	2.7 (1.0–5.9)^c^	0.81 (0.31–2.10)	0.364	0.52 (0.14–1.98)	0.34
Dementia present before admission	46 (44)^b^	23 (22)^b^	4.28 (1.88–9.76)	0.001	5.99 (1.86–19.24)	0.003
Most recent MMSE^d^	24 (21–27)^c^	27 (22–28)^c^	0.92 (0.86–0.98)	0.015	1.05 (0.95–1.16)	0.36
Cause of admission						
Infection	49 (47)^b^	18 (17)^b^	5.45 (2.00–14.89)	0.001	7.88 (1.61–38.49)	0.01
Other medical	37 (35)	50 (48)	Reference		Reference	
Fracture	15 (14)	5 (5)	5.66 (1.47–21.77)	0.01	7.36 (2.27–23.87)	0.001
Other surgical	4 (4)	32 (30)	0.17 (0.04–0.77)	0.02	0.16 (0.03–0.93)	0.04
Emergency (vs elective) admission	100 (95)^b^	82 (78)^b^	7.00 (2.09–23.47)	0.002	7.36 (1.98–27.35)	0.003
CRP (mg/L)^d^	16 (10–57)^c^	17 (10–43)^c^	1.00 (1.00–1.01)	0.86	1.00 (0.99–1.01)	0.79
Albumin (mg/L)^d^	40 (37–43)^c^	40 (36–43)^c^	0.98 (0.92–1.05)	0.55	1.03 (0.94–1.12)	0.54

^a^Mean (SD).

^b^N (%).

^c^Median (IQR).

^d^N missing data: Most recent MMSE, delirium = 7, not delirium = 4; CRP, delirium = 2, not delirium = 19; Albumin, delirium = 1, not delirium = 16.

### Outcomes after delirium

**Fig. 3:**
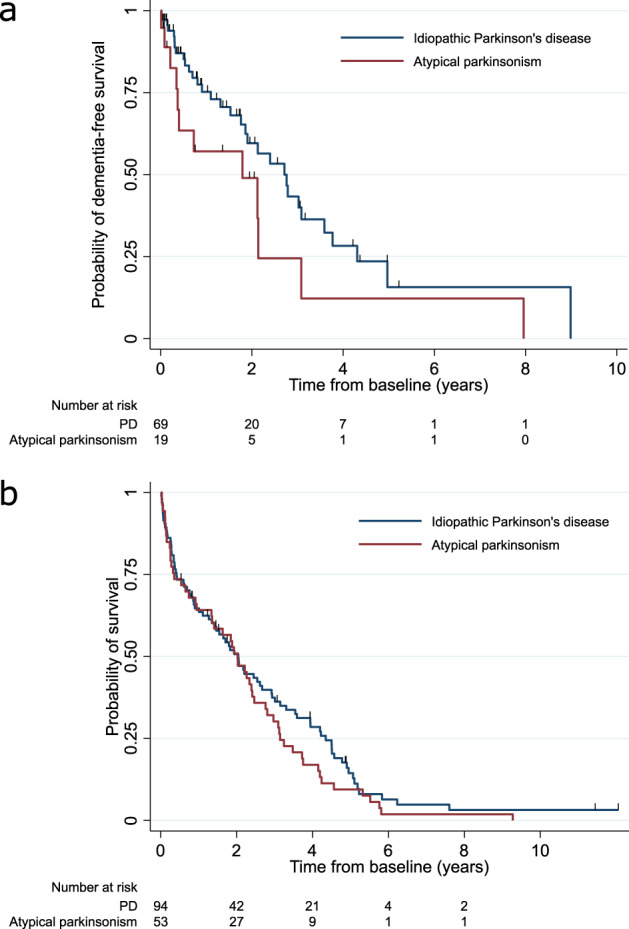
Outcomes after delirium. **a** Kaplan–Meier probabilities of dementia-free survival after delirium in PD and atypical parkinsonism in those without dementia at time of delirium. **b** Kaplan–Meier probabilities of survival after delirium in PD and atypical parkinsonism.

179 patients developed dementia (90 PD, 89 atypical parkinsonism) and 269 patients died (154 PD, 115 atypical parkinsonism) during follow-up (Table 6). Median time from delirium to dementia in those without dementia at the time of delirium in PD was 2.8 years (95% CI 1.9–3.6) and in atypical parkinsonism was 1.8 years (95% CI 0.34–3.1) (see Fig. 3a). Median time from delirium to death in all patients with delirium was 2.0 years (95% CI 1.5–2.4) with no difference between PD and atypical parkinsonism (see Fig. 3b). 50 patients with atypical parkinsonism had dementia at the time of diagnosis and were excluded from the analysis of the effect of delirium on developing dementia. The hazards of developing dementia after delirium (development of delirium as time-varying covariate) in PD and atypical parkinsonism were increased (HR = 6.93, 95%CI 4.18–11.48). There was no evidence that this differed between PD and atypical parkinsonism (*p*-value for interaction 0.72). Hazards of death were increased after delirium with evidence the HR varied with diagnosis (interaction *p* = 0.002): the HR in PD was 3.76 (95%CI 2.65–5.35) and in atypical parkinsonism was 1.59 (95% CI 1.04–2.42).

**Table 6:** Effect of delirium on dementia and death.

Variable	Dementia *N* = 239 (PD = 189, atypical parkinsonism = 50)^a^		Death *N* = 289 (PD = 190, atypical parkinsonism = 100)	
	Hazard ratio (95% CI)	*P*-value	Hazard ratio (95% CI)	*P*-value
Period after delirium versus before delirium				
All patients	6.93 (4.18–11.49)	<0.001		
In PD^b^			3.76 (2.65–5.35)	<0.001
In atypical parkinsonism^b^			1.59 (1.04–242)	0.03
Atypical parkinsonism vs PD	1.45 (0.77–2.75)	0.25	3.64 (2.40–5.52)	<0.001
Age at baseline (10-year increase)	1.66 (1.20–2.31)	0.002	1.69 (1.39–2.05)	<0.001
Sex	1.19 (0.71–1.98)	0.51	1.90 (1.44–2.52)	<0.001
DepCat	1.05 (0.89–1.23)	0.58	1.09 (1.00–1.18)	0.05
Baseline Charlson score	1.09 (0.91–1.31)	0.35	1.15 (1.06–1.25)	0.001
Baseline UPDRS part 3 (10-point increase)	1.12 (0.87–1.44)	0.39	1.26 (1.11–1.44)	0.001
Baseline MMSE	0.95 (0.87–1.04)	0.25	0.98 (0.94–1.02)	0.34

Mulitvariable Cox regression with time-varying covariate.

^a^50 patients with atypical parkinsonism were excluded because they developed dementia before entry into the study.

^b^Hazard ratios for the association between death and period before versus after delirium is presented separately for PD and atypical parkinsonism because a significant interaction between period and diagnosis was included in the model.

*DepCat* deprivation score, *MMSE* mini-mental state examination, *UPDRS* Unified Parkinson’s Disease Rating Scale.

## Discussion

This is the first study of the incidence of first hospital episode of delirium during the disease course of PD and atypical parkinsonism, and has shown that it is common in both, with the incidence in atypical parkinsonism more than double that in PD. Risk factors for delirium included atypical parkinsonism, higher age at diagnosis in PD, lower baseline MMSE, and the presence of the *APOE* ε4 allele and the H1/H1 *MAPT* haplotype. Developing dementia was associated with a doubling of the risk of delirium and was an important factor in the higher incidence in atypical parkinsonism than in PD. At the time of admission to the hospital, the causes of admission, emergency admission, and presence of dementia were significant predictors of delirium. Additionally, we demonstrated that delirium is associated with a high risk of developing dementia and mortality: after the development of delirium, there was a seven-fold increased risk of dementia and almost four-fold increased risk of death in PD versus before delirium.

There were differences in the risk factor profile between PD and atypical parkinsonism. There was a strong interaction between age and diagnosis with age being strongly associated with delirium in PD but not associated with atypical parkinsonism. Although the interaction with UPDRS was not significant, there was some evidence of a differential association with UPDRS in that this was associated with delirium in the model of PD with genetic markers but not in the main model. These risk factors are therefore less relevant in atypical parkinsonism than in PD which may be due to lower brain reserve in atypical parkinsonism such that other factors have less importance. Conversely, MMSE was associated in the main model (PD and atypical parkinsonism), but not in the model of PD only, with genetic markers, perhaps because PD has less cognitive impairment at baseline, so MMSE is less discriminatory in this group.

This is the first study to describe the development of delirium in PD over the disease course from diagnosis. Although no previous studies are directly comparable, our findings further support previous research which suggested that delirium is common in PD^2^. Data on the prevalence of delirium is difficult to compare with our incidence data. Two small studies have studied the prevalence of delirium in general hospital admissions in people with PD. A Dutch study of 46 participants (definition of delirium not stated) found a prevalence of 24% and a UK study of 53 admissions (using a DSM-5 definition) found a prevalence of 34%^5,9^. Several other studies found delirium to be common in specific settings including nursing homes^10^ and after DBS^6^. By comparison, the delirium point-prevalence across different inpatient settings in general (non-parkinsonian) populations ranges from 9-32%^11^.

Few studies have investigated delirium over the life course of any other neurodegenerative disorders. Lerner et al^12^ found that 22% of a community-dwelling sample of Alzheimer’s dementia had delirium during the course of their illness. Fong et al.^13^ studied delirium in an Alzheimer’s disease patient registry and found that delirium led to accelerated cognitive decline. However, they were unable to define its incidence because they only identified delirium episodes in patients admitted to one hospital but not others their patients were admitted to.

Few previous studies have investigated predictors of delirium in PD. Lawson et al found that older age, higher frailty scores, and longer hospital stay were associated with delirium in PD^5^. Carlson et al. found that previous history of delirium, older age, and disease duration were associated with delirium in PD following DBS insertion^6^. Evidence from studies in older people, in general, have also found that age and dementia increase the risk of delirium^14^. We did not find an association between CRP or albumin and delirium as has been demonstrated in previous studies^14,15^, which may be because the first measurement in an admission may be less predictive than later measurements, that these associations vary by setting, or because of lack of power.

Our data on genetic predictors of delirium in PD are novel: no previous studies, to our knowledge have previously identified polymorphisms which influence delirium risk in PD. Our finding of an association of the *APOE* ε4 allele with delirium accords with previous literature on its association with dementia^16^ and with some papers demonstrating an association with delirium severity in older adults^17^, although the evidence for its association with the occurrence of delirium is unclear^18^. Recent research demonstrating the *APOE* alleles modify the relationship between CRP and post-operative delirium suggests there may be a more complex relationship between *APOE* and delirium^19^. No previous studies have examined an association between *MAPT* variants and delirium but the H1/H1 haplotype has been associated with dementia in PD in some studies^20^ though not others^21^ so further studies or meta-analysis of these studies is needed. Although GBA variants have been associated with dementia in PD^22^, we did not find an association with delirium. It is possible this study was underpowered to find a weak association. Analysis of the *COMT* Val^158^Met polymorphism and its association with delirium would be useful to extend this work as this has previously been associated with delirium after head injury^23^ although it has been reported to have no association with dementia in PD^24^.

We also identified that delirium is more common in atypical syndromes, after adjustment for age and other potential confounders, which may reflect disease-specific factors affecting cognitive reserve. The only study we have identified with data on delirium in atypical parkinsonism was a study reporting the prevalence of delirium in consecutive patients with DLB in a memory clinic in Japan (using the DSM-IV diagnostic criteria to define delirium) to be 32%^8^.

Our finding of an increased risk of developing dementia and mortality after delirium has been suggested previously in one very small study of only 21 PD patients^7^. This finding is consistent with evidence from several studies of faster cognitive decline and increased dementia risk and mortality after delirium in the general population^25–27^. We found that dementia did not necessarily quickly follow delirium. The median time to dementia after delirium was nearly 3 years in PD, and some did not develop dementia for substantially longer than this.

This study has several key strengths. The use of an incidence-based inception cohort with a high consent rate to follow-up, and low attrition rates lead to a low risk of selection bias and high generalisability to the general parkinsonism population. Moreover, few patients migrated out of the study area, a single volume of case notes were used across both general hospitals in the study area, case notes were available from the vast majority of patients, and case notes were comprehensively reviewed so we believe we have comprehensive data on hospital episodes of delirium. Furthermore, we used, previously validated definition of delirium. These criteria have a better balance of sensitivity and specificity than either the “probable delirium” or “possible delirium” as defined by the chart abstraction method published by Kuhn et al.^28^. Furthermore, our finding that all those with a documented diagnosis of delirium by an expert clinician met the criteria provides further validation of its sensitivity, although these cases may not be representative of all cases of delirium. We also used a clinical diagnosis of dementia, rather than basing our diagnosis on cognitive scores alone.

Nevertheless, this study also has limitations. The main limitation is that we used a retrospective chart review to assess delirium, so a degree of misclassification was inevitable. The validation study of the retrospective chart method that we used demonstrated 73% sensitivity and 83% specificity versus a prospective assessment with daily evaluation including cognitive assessment^29^. Therefore, under-ascertainment was probably more common, possibly because in some cases delirium symptoms were not documented in patients’ notes, although we sought to minimise this by using all available records, including nursing records. This misclassification may be more likely in cases with dementia because of similar symptoms in delirium and dementia. Prospective studies of delirium in parkinsonism are required but will be labour intensive, particularly if community and hospital-based episodes are to be identified. We have only explored hospital-based delirium but this is likely to identify most of the more-severe delirium episodes. Community episodes of delirium in patients not admitted to hospital were not identified. This would be a useful area for future research, but any such study would require substantial resources to review primary care records. We did not have data on several factors which have previously been associated with delirium in the older population, including frailty, sensory impairment, alcohol intake, and nutritional status^14,30^. Another limitation relates to the lack of control data on delirium (due to insufficient resources to look at this in the PINE study controls) which, if available, would have allowed measurement of the excess risk of delirium in parkinsonism over controls. Another limitation relates to the correct identification of the timing of dementia. Because we usually only formally assessed participants for dementia annually, some patients in early stages of dementia with delirium may have been misclassified as being free of dementia. This bias may lead to over-estimation of the association between delirium and later dementia but is less likely to affect the association between dementia and later delirium. Furthermore, the associations between delirium and outcomes may be confounded by the number of hospital admissions, as we would have been more likely to identify delirium in those with more frequent hospital admissions, who are also more likely to have poorer outcomes. However, as delirium was sometimes the cause of hospital admissions, adjusting for this may introduce another bias in the estimation of these associations. Even if confounding by the number of hospital admissions is present, this does not alter the prognostic value of the presence of hospital delirium episodes. We did not have data on delirium episodes prior to diagnosis. Lastly, we did not have sufficient power to distinguish between atypical parkinsonian syndromes in our analyses. It would be useful to examine whether multiple system atrophy has a lower frequency of delirium than other syndromes, for instance.

This work demonstrates the importance of delirium in PD and atypical parkinsonism, an under-researched non-motor feature of these disorders. It is evident that delirium in parkinsonism is an area requiring further research, specifically into (i) its risk factors, both factors which change over time from diagnosis and further genetic predictors; (ii) other key outcomes including falls, fractures and institutionalisation; and (iii) in individual atypical syndromes where little is known about delirium.

Given its poor outcomes, prevention of, early identification of, and management of delirium is important. At present, we lack clear evidence that treatments of delirium lead to better outcomes so further research is required^31–33^.

## Methods

### The PINE study

The Parkinsonism Incidence in North-East Scotland (PINE) study is a prospective, community‐based incidence cohort of parkinsonism in Aberdeen, UK. We sought to identify all new cases of degenerative and vascular parkinsonism over a total of 4.5 years between 2002-4 and 2006-9 in Aberdeen, UK, using multiple overlapping methods of case ascertainment, as previously described^34–36^. Patients thought to be parkinsonian were recruited at disease inception and followed life-long with annual expert review of diagnoses (guided by the UK Brain Bank Criteria for PD and formal criteria for other syndromes)^37–40^. At each visit data collected included Mini-Mental State Examination (MMSE), Charlson co-morbidity score^41^ and Unified Parkinson’s Disease Rating Score (UPDRS). At each visit, the examining doctor assessed whether the patient had developed dementia. We used a clinical definition of dementia: progressive impairment in multiple cognitive domains, impacting upon daily activities, with decline in functioning, not exclusively caused by delirium. This closely follows the DSM-IV criteria^42^ with the exception that memory impairment did not have to be present, provided there were multiple other cognitive domains impaired. We did not use the Movement Disorders Task Force criteria^43^ because we had not collected data in all our patients on all the tests required for either Level I or Level II testing (the PINE study predated its publication). Genotyping of microtubule-associated protein tau [*MAPT*] H1 versus H2 haplotype, Apolipoprotein E [*APOE*], and glucocerebrosidase [*GBA*] gene variants) were available from a proportion of patients with PD. Informed consent was obtained for recruitment and follow-up, including of review hospital case notes. Patients were linked to the NHS central register for routine notification of deaths.

For the present study we performed retrospective case note review to identify the first hospital delirium episode in patients among patients who were under follow-up in the PINE study. We included patients with PD and atypical parkinsonism (progressive supranuclear palsy, multiple system atrophy, dementia with Lewy bodies, vascular parkinsonism, corticobasal degeneration or dementia with associated parkinsonism). All available hospital case notes were reviewed including medical and nursing notes. Only two secondary care hospitals served the study population, which received all acute medical and surgical admissions in the area. The same case notes were used across both hospitals. The period of case note review was from diagnosis of parkinsonism until the end of 2018.

### Definition of delirium

The diagnosis of delirium was adapted from a validated chart-based method described by Inouye et al^29^ that was chosen for its published validation with high sensitivity and specificity. We defined delirium by the presence of all three of the following criteria:

1. Terms indicating altered mental status such as delirium, mental state change, inattention, disorientation, hallucinations, agitation, inappropriate behaviours, confusion.
2. Evidence of acute onset (e.g. a statement that these symptoms were not present before current illness; statement indicating normal baseline cognition; or statement indicating that cognition worse than normal).
3. Evidence of fluctuating course (e.g. explicit description of fluctuation or statements indicating that the patient’s mental status was worse at one time and better at another time).

Alternatively, a documented diagnosis of delirium made by a geriatrician, neurologist or psychiatrist was also sufficient to make a diagnosis of delirium. We did not distinguish whether terms indicating altered mental status related to delirium or to the underlying parkinsonian disorder because the second criterion (acute onset) would not be met if the altered mental status was just due to the underlying parkinsonian disorder. Date of delirium was defined as the date of admission to hospital during which delirium occurred. We also documented the main cause of delirium.

### Incidence of first delirium episode

The incidence rate of first delirium episode was calculated per 100 person years (number of new cases/person years follow-up free of delirium) with confidence intervals calculated assuming a Poisson distribution. Median time to delirium was based on Kaplan–Meier survival probabilities with patients censored at death or at end of period of case note review (31^st^ December 2018).

### Predictors of delirium

We used Cox regression to analyse baseline predictors of delirium (measured at recruitment to study, i.e., shortly after diagnosis of the parkinsonian syndrome). We first entered demographic and clinical variables which were previously associated with risk of delirium or poor outcomes in PD based on previous studies into a multivariable Cox model:^5,14,36,44^ diagnosis (PD or atypical parkinsonism), baseline age (i.e., measured at diagnosis), sex, DepCat (a postcode-based measure of socioeconomic status ranging from 1 [least deprived] to 6 [most deprived]), baseline MMSE, baseline Charlson score, and baseline UPDRS motor score. Other than sex, these variables were entered as continuous variables. We investigated an interaction between age and diagnosis and between UPDRS and diagnosis category. We performed a secondary analysis of prognostic factors in those without dementia by censoring patients at the time of dementia.

We also investigated genetic predictors of delirium in PD using genetic variants that have previously been associated with prognostic outcomes in PD, including dementia^20,22,45^. These were the presence of any *GBA* variant (available for the E326K (rs2230288), T369M (rs75548401), V460L (rs369068553), Y135C (rs781152868), N370S (rs76763715), and L444P (rs421016) variants), the presence of the *MAPT* H1/H1 haplotype, and *APOE* ε4 carrier versus non-ε4 carrier status. Because these data were only available in a subset with PD, we developed a second model in these patients with each variant entered as binary variables together with the demographic and clinical variables listed above.

We thirdly investigated the influence of dementia on the incidence of first delirium episode with the Cox model with the presence of dementia as a time-varying covariate, adjusted for demographic and clinical confounders. We tested an interaction between diagnosis (PD vs atypical parkinsonism) and the effect of dementia to test whether the effect of dementia on delirium varied by diagnosis.

### Predictors of delirium at time of hospital admission

We also investigated risk factors for delirium at the time of admission to the hospital using a nested case-control analysis. Cases were defined as patients admitted to hospital with delirium, and controls were patients admitted to hospital without delirium. Cases and controls were matched 1:1 with exact matching on sex, diagnosis (PD or atypical parkinsonism), year of follow-up in the study (disease duration) and with nearest-neighbour matching on age. We investigated the following risk factors for delirium: cause of hospital admission (infection, other medical cause, fracture, or other surgical cause); emergency versus elective admission; most recent MMSE; and serum parameters which have previously been identified in systematic reviews to be associated with delirium in acutely unwell patients: C-Reactive Protein (CRP) and albumin^14,15^. Where these blood tests were measured multiple times during an admission, we used the first measurement. Univariable and multivariable conditional logistic regression was used to calculate odds ratios (OR) and 95% confidence intervals (CI) for the associations between the risk factors and delirium adjusted for exact age and exact disease duration as these variables were not matched exactly.

### Outcomes after delirium

We plotted Kaplan–Meier probabilities of remaining (i) dementia-free and (ii) alive. Patients were censored at death (in the dementia analysis) or when last seen in those with ongoing follow-up. Hazards of these outcomes were explored using multivariable Cox models with the development of delirium as a time-varying covariate (i.e. delirium coded as 0 until time of development of delirium and as 1 thereafter). These models were adjusted for baseline age, sex, MMSE, UPDRS part 3, deprivation category, and Charlson score. Interactions between the time-varying delirium covariate and both age and diagnostic category (PD versus atypical parkinsonism) were explored. We did not use a competing risks analysis for the analysis of dementia as deaths prior to dementia were less likely to be directly related to PD and therefore mostly independent of the development of dementia.

The distribution of variables was assessed by inspecting histograms. The proportional hazards assumption was verified by visual inspection of Kaplan–Meier plots by levels of predictor variables. Because 9% of patients had missing values of baseline MMSE, which were assumed to be missing at random, we used multiple imputation to impute missing values of MMSE in each of these models of predictors and outcomes. We used a predictive mean matching algorithm to impute these data using all the variables in the model and two additional variables: the presence of cognitive symptoms at baseline and MMSE at one year of follow-up where available. For each model, 20 imputed datasets were combined using Rubin’s rules.

Statistical analysis were performed using Stata version 16.

### Ethical approval

Ethical approval was granted by the Multi-centre Research Ethics Committee for Scotland. Participants provided written informed consent for research or, if the participant lacked capacity due to dementia, assent to participation was provided by the next of kin.

### Reporting summary

Further information on research design is available in the Nature Research Reporting Summary linked to this article.

## Supplementary information

Supplementary Data 1

Reporting Summary

## Data Availability

The data are available on request from the authors.
